# Updated Taxonomy of *Pectobacterium* Genus in the CIRM-CFBP Bacterial Collection: When Newly Described Species Reveal “Old” Endemic Population

**DOI:** 10.3390/microorganisms8091441

**Published:** 2020-09-20

**Authors:** Perrine Portier, Jacques Pédron, Géraldine Taghouti, Cécile Dutrieux, Marie-Anne Barny

**Affiliations:** 1 IRHS-UMR1345, CIRM-CFBP, Institut Agro, INRAE, Université d’Angers, SFR 4207 QuaSav, 49071 Beaucouzé, France; perrine.portier@inrae.fr (P.P.); geraldine.taghouti@inrae.fr (G.T.); cecile.dutrieux@inrae.fr (C.D.); 2 Sorbonne Université, INRAE, Institute of Ecology and Environmental Sciences-Paris, 4 place Jussieu, F-75 252 Paris, France; jacques.pedron@upmc.fr

**Keywords:** *Pectobacterium*, collection, taxonomy, host plants

## Abstract

Bacterial collections are invaluable tools for microbiologists. However, their practical use is compromised by imprecise taxonomical assignation of bacterial strains. This is particularly true for soft rotting plant pathogens of the *Pectobacterium* genus. We analysed the taxonomic status of 265 *Pectobacterium* strains deposited at CIRM-CFBP collection from 1944 to 2020. This collection gathered *Pectobacterium* strains isolated in 27 countries from 32 plant species representing 17 botanical families or from nonhost environments. The MLSA approach completed by genomic analysis of 15 strains was performed to update the taxonomic status of these 265 strains. The results showed that the CIRM-CFBP *Pectobacterium* collection harboured at least one strain of each species, with the exception of *P. polonicum*. Yet, seven strains could not be assigned to any of the described species and may represent at least two new species. Surprisingly, *P. versatile*, recently described in 2019, is the most prevalent species among CIRM-CFBP strains. An analysis of *P. versatile* strains revealed that this species is pandemic and isolated from various host plants and environments. At the opposite, other species gathered strains isolated from only one botanical family or exclusively from a freshwater environment. Our work also revealed new host plants for several *Pectobacterium* spp.

## 1. Introduction

Bacterial collections are invaluable tools for microbiologists, as they host many strains isolated at different times on different hosts or environments and on different countries and continents. As such, they summarise the collective sampling and research efforts performed by bacteriologists from all over the world on many different bacterial species. This collective treasure is nevertheless often underexploited for several reasons, the main one being the poor taxonomical assignation of many deposited strains to current taxonomical standard. This situation results from the fact that many strains are ancient strains that were deposited in collection before the precise taxonomic delineation of species through genome analysis were performed. Even if most collections are doing great efforts to improve this situation, a lot of work is still necessary. Therefore, currently, collections harbour many strains with old names no longer reflecting their actual taxonomical status. Such ancient strains are nevertheless important to understand the epidemiology of a given species, when and where a particular species was first isolated in the world and what is its historical prevalence all over the world. 

Soft rot plant pathogenic bacteria of the *Pectobacterium* genus represent an archetype of this situation. They are characterised by their ability to degrade plant cell walls through the secretion of a cocktail of plant cell wall degrading enzymes (PCWDEs) [1,2]. *Pectobacterium* spp. are a major cause of harvest loss of potatoes both on the field and during potato storage. However, strains of this genus have also been collected on a large number of host plants and are thus known as large host range plant pathogens, although the extent of the host range varies between species [3,4]. *Pectobacterium* spp. were previously regrouped in the *Erwinia* genus founded in 1917 to unite all Gram-negative, fermentative, nonsporulating and peritrichous flagellated plant pathogenic bacteria [5]. Early taxonomy recognised three taxa within these soft rot bacteria: *Erwinia carotovora* subsp. *carotovora*, *Erwinia carotovora* subsp. *atroseptica* and *Erwinia chrysanthemi* [6,7] that were included in the Approved Lists of Bacterial Names in 1980 either under the species named *Erwinia* or *Pectobacterium* [8], and the *Pectobacterium* genus was formally described in 1998 [9]. In 2005, on the basis of the 16S RNA sequence, *P. chrysanthemi* was reclassified within the new *Dickeya* genus [10]. Furthermore, in addition to *Pectobacterium carotovorum* subsp. *carotovorum* and *Pectobacterium carotovorum* subsp. *atrosepticum*, several new subspecies were progressively described for *P. carotovorum*: *Pectobacterium carotovorum* subsp. *brasiliense* [11,12], *Pectobacterium carotovorum* subsp. *wasabiae*, *Pectobacterium carotovorum* subsp. *betavasculorum*, *Pectobacterium carotovorum* subsp. *odoriferum* [9] and *P. carotovorum* subsp. *actinidiae* [13]. All these subspecies were latter elevated to species level [14,15] following genomic analysis [16]. In addition, new *Pectobacterium* species were progressively described, most of them recently on the basis of whole-genome sequence analysis. Today, the *Pectobacterium* genus encompasses 17 recognised species: *Pectobacterium actinidiae* [15], *Pectobacterium aquaticum* [17], *Pectobacterium aroidearum* [18], *Pectobacterium atrosepticum* [14], *Pectobacterium betavasculorum* [14], *Pectobacterium brasiliense* [15], *Pectobacterium cacticida* [9,19], *Pectobacterium carotovorum* [8,15,20], *Pectobacterium fontis* [21], *Pectobacterium odoriferum* [15], *Pectobacterium parmentieri* [22], *Pectobacterium parvum* [23], *Pectobacterium polaris* [24], *Pectobacterium polonicum* [25], *Pectobacterium punjabense* [26], *Pectobacterium versatile* [15] and *Pectobacterium wasabiae* [14] and two proposed species not yet validated by ad hoc committees: “*Pectobacterium peruviense*” and “*Pectobacterium zantedeschiae”* [27,28]. Given the taxonomic evolution in the past ten years, the ecological importance, repartition and habitat of most species need to be evaluated. Bacterial collections are interesting tools to reach that goal. 

The CIRM-CFBP, French Collection for Plant-associated Bacteria (DOI: 10.15454/1.5103266699001077E12) located in France at INRAE, hosts many strains of the *Pectobacterium* genus isolated from 1944 to 2019. However, the taxonomical status of most of these strains is unclear. Many strains were deposited as *Pectobacterium* spp., which indicates they belong to this genus. Furthermore, other strains were deposited as *P. carotovorum.* Since, historically, *P. carotovorum* has gathered seven subspecies that are now elevated to species level, it is currently difficult to know to which species these strains belong. Finally, many ancient strains were likely characterised solely on the basis of phenotypic tests, and this could have led to incorrect taxonomic assignation. As a result, it is currently impossible to have a synthetic view of the *Pectobacterium* collection hosted at the CIRM-CFBP. 

The aim of this work was to clarify and update the taxonomic status of 265 *Pectobacterium* strains deposited at the CIRM-CFBP collection and to gain insight of the frequency and isolation habitat of the 19 described *Pectobacterium* species within the CIRM-CFBP collection. To do so, we performed a phylogenetic analysis based on the partial sequences or *dnaX*, *leuS* and *recA* housekeeping genes that allocated strains to specific clades. To understand how clades were related to delineate species, this analysis was completed with the genome sequencing of 15 strains. This allowed determining the frequency of each species within the CIRM-CFBP collection. Some species appeared to be pandemic *Pectobacterium* species found all over the world on various host plants and environments, while others, at the opposite, gathered strains isolated from only one botanical family or one specific environment. 

## 2. Materials and Methods

### 2.1. Bacterial Strains, Culture Conditions and DNA Extraction

The 265 strains used in this study are provided Table S1. For housekeeping genes amplification, PCRs were conducted directly on colonies grown overnight on solid King B medium (2-g protease peptone, 15-g agar, 10-mL glycerol, 1.5-g KH_2_PO_4_ and 1.5-g MgSO_4_-7H_2_O per one litre of medium) and boiled at 100 °C for 10 min. For the preparation of genomic DNA, the strains were first grown overnight at 28 °C on solid LB medium (10-g tryptone, 5-g yeast extract, 10-g NaCl and 15-g agar per one litre of medium). A single colony was then picked up and grown overnight in 2 mL of liquid LB medium at 28 °C agitated at 120 rpm. After centrifugation of the culture broth (5 min at 12,000 rpm), DNA was extracted with the wizard^®^ genomic DNA extraction kit (Promega, Madison, WI, USA) following the supplier’s instructions. DNA was suspended in 100 μL of sterile distilled water, and the quantity and quality of DNA was assessed by NanoDrop measurement, spectrophotometry analysis and agarose gel electrophoresis on 1% agarose gels. 

### 2.2. dnaX-leuS-recA Phylogeny

Housekeeping genes *dnaX leuS* and *recA* were amplified and sequenced for the 261 strains. PCR protocols and primers were described in Portier et al. [15]. PCR products sequencing was performed by Genoscreen (Lille, France). The consensus sequences for each gene for each strain were extracted from forward and reverse sequence assemblies using Geneious Pro version 9.1.8 (www.geneious.com). The sequences were then aligned and trimmed using BioEdit version 5.0.6. All the obtained sequences were deposited in public databases, and Table S1 summarises the data. A phylogenetic tree was constructed with concatenated alignments of all genes with MEGA 7.0.26 using the neighbour-joining method with 1000 bootstrap replicates, and the evolutionary distances were computed by using the Kimura two-parameter method. All the *Pectobacterium* type strains were included in the phylogenetic tree. When type strains were not present at CIRM-CFBP (*P. polonicum* DPMP 315^T^, *P. actinidiae* KKH3^T^, *P. zantedeschiae* 9M^T^ and *P. peruviense* UGC32^T^), *dnaX, leuS* and *recA* sequences were retrieved from the genome sequences available at NCBI. In addition, to help species delineation on the phylogenetic tree, *dnaX, leuS* and *recA* sequences retrieved of other NCBI genomes recently reclassified in their correct assignation species by Portier et al. [15] were also included. To root the phylogenetic tree, *dnaX, leuS* and *recA* sequences were retrieved from the genome of *Dickeya solani* CFBP 7704 (RNS 08.23). Twelve strains were not included in the phylogenetic analysis provided in Figure S1, because at least one of the three sequences of *dnaX, leuS* or *recA* was not correctly amplified. However, for these 12 strains, the remaining amplified sequences (data not shown) allowed their assignation to known species without ambiguities. Finally, 4 strains (CFBP 8719, CFBP 8720, CFBP 8723 and CFBP 8724) deposited at the CIRM-CFBP in 2019 were assigned to their species following amplification and sequencing of the *gap*A housekeeping gene as described by Cigna et al. [29]. All the sequences used for the phylogenetic tree were deposited at NCBI, and the accession numbers are listed in Table S1. 

### 2.3. Genome Analysis

Genome sequencing was performed at the next-generation sequencing core facilities of the Institute for Integrative Biology of the Cell (91190 Gif-sur-Yvette, Avenue de la Terrasse, France). Nextera DNA libraries were prepared from 50 ng of high-quality genomic DNA. Paired-end 2 × 75-pb sequencing was performed on an Illumina NextSeq 500 apparatus, with a High Output 150 cycle kit. CLC Genomics Workbench (Version 9.5.2, Qiagen Bioinformatics) was used to assemble reads. Final sequencing coverage was between 118× and 216× (Table 1). Coding sequences were predicted using the RAST server [30] with the Glimmer 3 prediction tool [31]. Statistics of the 15 newly sequenced draft genomes are presented in Table 1.

Pairwise comparison of the genomes was computed using the average nucleotide identity (ANI) Pyani python module (https://github.com/widdowquinn/pyani) [32] with the blast algorithm (ANIb). The species threshold was set at 96%. Digital DNA-DNA hybridisation (dDDH) values were calculated between each sequenced genome and reference species genomes using a dedicated pipeline (http://ggdc.dsmz.de/) from the formula 2 (sum of all identities found in high-scoring segment pairs (HSPs) divided by overall HSP length); this measure is normalised to the genome length and, therefore, is still robust when incomplete draft genomes are analysed. The species threshold was set to 70%.

A phylogenetic tree was constructed from concatenated sequences of 1053 homologous genes retrieved from the 15 newly sequenced genomes and 18 genomes of type strains or reference strains for each species. MLSA analysis was performed as described in Portier et al. [15]. The genome of *D. solani* strain RNS_08.23 was used to root the tree. The species *P. cacticida* was not included in this analysis, as no reference genome has yet been published.

## 3. Results and Discussion

### 3.1. The CIRM-CFBP Studied Strains 

**Figure 1:**
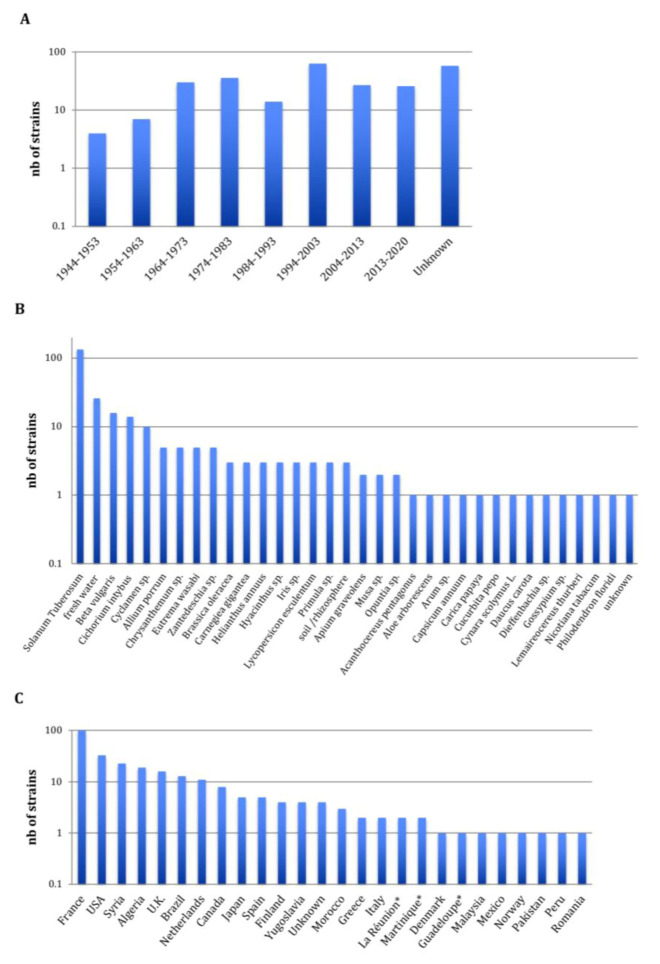
Overview of the 265 CIRM-CFBP *Pectobacterium* strains at the beginning of our study. (**A**) Decade of isolation, (**B**) host or environment from which the strains were isolated and (**C**) country from which the strains were isolated; the stars indicate French overseas territories. Complete details for strains are available in Table S1.

The 265 *Pectobacterium* CIRM-CFBP-studied strains are presented in Figure 1. They were isolated from 1944 to 2019 covering, therefore, a 75-year period. For 56 strains, the year of isolation was not reported; however, for these strains the year of the deposit at the CIRM-CFBP indicated that 22 strains were isolated at least 46 years ago (deposited from 1970 to 1974), 1 strain was isolated at least 38 years ago (deposited in 1982), 6 strains were isolated at least 28 years ago (deposited from 1991 to 1992) and 28 strains were isolated at least 19 years ago (deposited in 2001) (Figure 1A). Concerning the host plants or environments from which these 265 strains were isolated, a large majority of 136 strains were isolated from potato tubers or potato plants, accounting for the threat provoked by *Pectobacterium* spp. on this economically important crop plant (Figure 1B) [4]. In addition, the studied collection gathered 100 strains isolated from 31 host plants covering 17 botanical families, accounting for the broad host range of *Pectobacterium* spp. [3], as well as 29 strains isolated from freshwater, soil or rhizosphere. As the CIRM-CFBP collection is a French collection, it is not surprising that a large majority of 100 strains originated from this country (Figure 1C). Fours strains originating from overseas French territories (Martinique, Guadeloupe and La Réunion) were considered apart, as these territories are located respectively on the North American continent (Martinique and Guadeloupe) and African continent (La Réunion). Overall, the collection gathers strains originating from 27 countries in Europe, Africa, North America, South America, Asia and Indonesia. Finally, 94 strains were deposited at the CIRM-CFBP as *Pectobacterium* spp., without any indication of the species they belong. Furthermore, many strains were deposited under names that no longer exist or under the *P. carotovorum* name, which could be misleading, since it previously gathered several subspecies. In summary, although this collection gathers many strains sampled at different times over different countries and environments, its practical use is hampered by the poor taxonomic designation of the deposited strains.

### 3.2. dnaX-leuS-recA Phylogeny of the Collection

**Figure 2:**
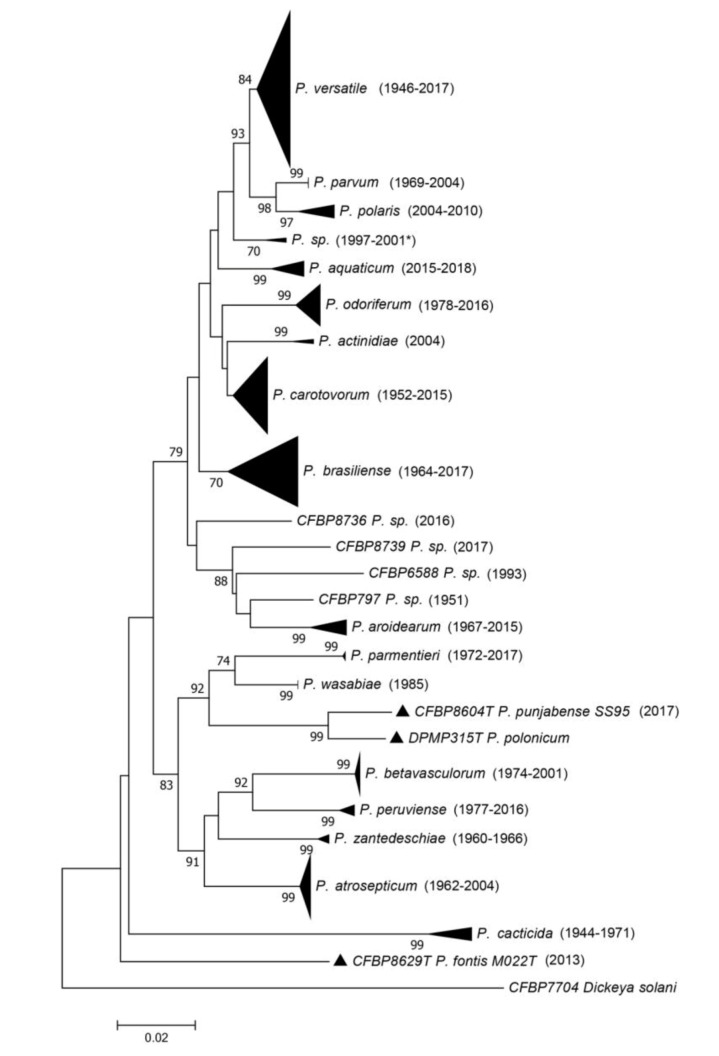
Phylogenetic tree reconstructed from concatenated partial sequences from *dnaX*, *leuS* and *recA* housekeeping genes. The phylogenetic tree was reconstructed with concatenated alignments of all genes with MEGA 7.0.26 using the neighbour-joining method with 1000 bootstrap replicates, and the evolutionary distances were computed by using the Kimura two-parameter method. Bootstrap values are shown when over 70. Between parentheses are indicated the earliest and latest isolation years for each species. When there is only one strain in the clade, the year of isolation for that strain is indicated. For one clade, the latest year of isolation is unknown, and the latest year of deposit is indicated as follows: 2001*. Full view of this tree and the accession numbers of the sequences are available in Figure S1 and Table S1, respectively.

The *dnaX-leuS-recA* phylogeny (Figure 2 and Figure S1) revealed that most strains spread out in clades that are separated from each other’s and supported by usually high bootstrap values. As most of these clades included the type strain of a given species, this allowed assigning most of the strains of CIRM-CFBP to 18 of the 19 described species within *Pectobacterium*, except *P. polonicum*. 

The relative position of the *P. caticida* species inside the *Pectobacterium* genus remains questionable, since no genome of this species has been sequenced, and, at the time of the description in 1991 by Alcorn et al. [19], most of the *Pectobacterium* species were not yet described. Interestingly, the *leuS, recA* and *dnaX* phylogeny performed here grouped all the *P. cacticida* strains in the same clade as a deep branching species within the *Pectobacterium* genus (Figure 2). 

For seven strains (CFBP 797, CFBP 5380, CFBP 6067, CFBP 6168, CFBP 6588, CFBP 8736 and CFBP 8739), the assignation to an already described species was either not possible or their position on the phylogenetic tree was ambiguous (Figure 2). These seven strains may belong to yet undescribed species; however, the *dnaX-leuS-recA* phylogeny performed here is not adequate to delineate new species. Three of these strains grouped together in the same *dnaX-leuS-recA* clade and may represent a single species (two are displayed in the phylogenetic tree in Figure S1), while the four remaining strains each represent a different clade. We sequenced the genomes of two strains out of these seven strains. Furthermore, in order to check that large clades revealed by the *dnaX-leuS-recA* phylogeny indeed represented a single species, we sequenced 13 genomes to complete this analysis. 

### 3.3. Whole-Genome Strains Analysis

**Figure 3:**
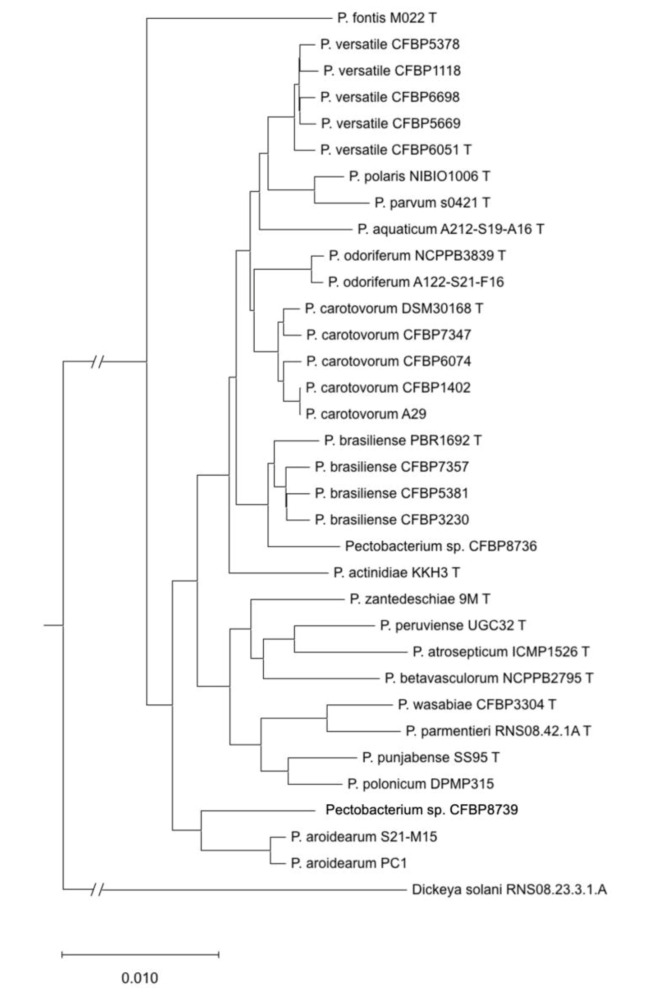
Phylogenetic tree reconstructed from the concatenated sequences of 1053 homologous gene sequences retrieved from complete genome sequences for the 15 sequenced strains and type strains or reference strains of other species.

A phylogenetic tree constructed from concatenated sequences of 1053 homologous genes retrieved from the 15 analysed genomes and the genomes of type strain or representative species is presented in Figure 3. Pairwise ANIb and dDDH were performed between the genomes of these 15 strains and the genomes of type strains or representative species. The results of ANIb and dDDH (Table S2) allowed to classify all the newly sequenced strains but two to already described species: four of the sequenced strains were classified as *P. carotovorum*, four as *P. versatile*, three as *P. brasiliense*, one as *P. odoriferum* and one as *P. aroidearum*. 

Strain CFBP 8736 displays pairwise ANI and DDH values below the species threshold with the *P. brasiliense* type strain (ANI: 94.7%; DDH 61.1%) (Table 2). However, the pairwise ANI/DDH values are higher (95.5% to 95.7% and 64.7% to 65.3%, respectively) between CFBP 8736 and the other *P. brasiliense* genomes (Table 2). The *P. brasiliense* species shows a relatively high level of divergence between its strains and is probably ongoing a diversification process [15]. Our results show that strain CFBP 8736 belongs to a separate species but is very close to *P. brasiliense*.

The remaining genome, corresponding to strain CFBP 8739, could not be assigned to known or proposed species. Analysis of the pairwise ANI/dDDH values obtained for this latter genome with type strains or reference strains of other species indicated that its closest species was *P. aroidearum*, with a pairwise ANI value of 91.1% and dDDH value of 43.2%, well below the cut-off values of the species limit (Table 2). Strain CFBP 8739 therefore belongs to a still-uncharacterised species. Its position in the *dnaX*-*leu*S-*recA* phylogenetic tree was distinct but close to two other strains, CFBP 6588 and CFBP 797, which could not be assigned to any known species (Figure 2 and Figure S1). However, it remains ambiguous to decipher if CFBP 6588 and CFBP 797 could be grouped either with *P. aroidearum* or with this new species represented by strain CFBP 8739 or if each strain represents a new species. Interestingly, these three strains were isolated from various environments: a monocot (*musa* sp. for CFBP6588), a dicot (*nicotiana tabacum* for CFBP 797) and freshwater (for CFBP 8739).

### 3.4. Comparison of the Updated Taxonomy with the Former One

**Figure 4:**
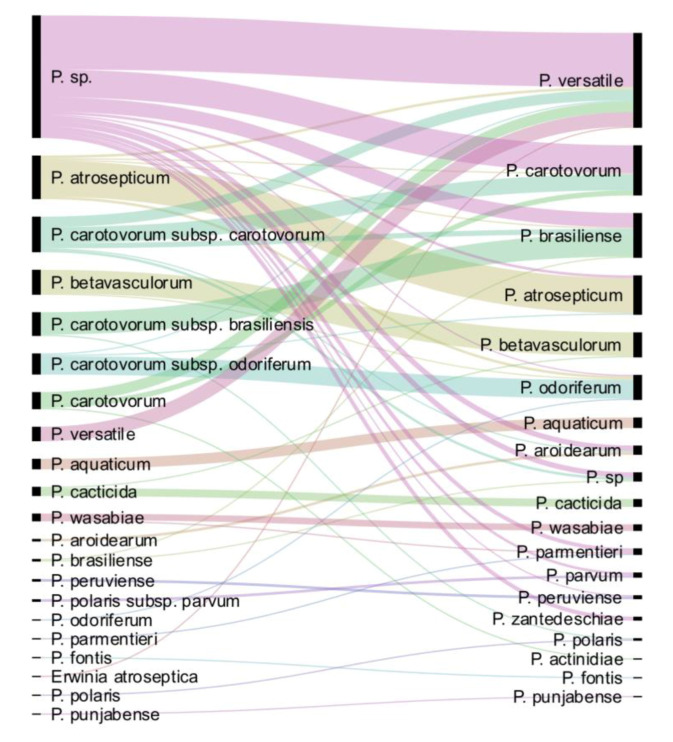
Comparison of the updated taxonomy with the former one. On the left are displayed the taxonomic names under which the strains were deposited; on the right, the updated taxonomy for the 265 strains is displayed.

Following *dnaX-leuS-recA* phylogeny and genome analysis, out of the 265 strains analysed, 157 strains could be assigned to 18 known or proposed species. The only species not represented in the CIRM-CFBP collection is *P. polonicum.* We performed a comparison of the updated taxonomy with the former one (Figure 4). Among the 94 formerly unassigned *Pectobacterium* sp. strains, 90 were taxonomically assigned to 10 different species. The more frequently assigned species were *P. versatile* (41/94 strains), *P. carotovorum* (21/94 strains) and *P. brasiliense* (11/94). Interestingly, three strains of formerly unassigned *Pectobacterium* sp. were finally assigned to *P. zantedeschiae*, a species without known representative in the CIRM-CFBP collection before our work. Most of the 27 former *P. carotovorum* subsp. *carotovorum* strains were split between *P. carotovorum* (12/27) and *P. versatile* (8/27). As well, the 13 strains previously classified as *P. carotovorum* were mostly split between *P. versatile* (8/13) and *P. carotovorum* (4/13). This highlights the close proximity between *P. versatile* and *P. carotovorum,* as already noted [15]. Conversely, strains assigned to the former *P. carotovorum* subsp. *brasiliense* and *P. carotovorum* subsp. *odoriferum* were mostly assigned to their cognate species *P. brasiliense* (16/17) and *P. odoriferum* (14/16) (Figure 4). As well, strains assigned to the species *P. atrosepticum* (27/33), *P. betavasculorum* (18/19), *P. aquaticum* (8/8), *P. cacticida* (6/7) and *P. wasabiae* (5/6) remained mostly associated to the same species, indicating former good taxonomic resolution of these groups. Finally, while the former classification assigned only two strains to the *P. aroidearum* species, this species was enriched of five strains following our taxonomical update. Out of these five strains, four were previously designated as *Pectobacterium* sp. and one as *P. carotovorum*.

Interestingly, seven strains could not be assigned to any known or proposed species. Three strains (CFBP 5380, CFBP 6067 and CFBP 6068, the latter not displayed in the phylogenetic tree; Figure S1) could probably be gathered in the same new species, since they are closely clustered in the same clade following *dnaX-leuS-recA* analysis. It remains unclear how many different species could be described with the four remaining strains. Among these, CFBP 8736 and CFBP 8739, whose genomes were sequenced, represent potentially two new species close to *P. brasiliense* and *P. aroidearum*, respectively. These two strains were isolated from river water in France in 2016 and 2017. 

### 3.5. Analysis of Strains Isolated on Potato Plants

**Figure 5:**
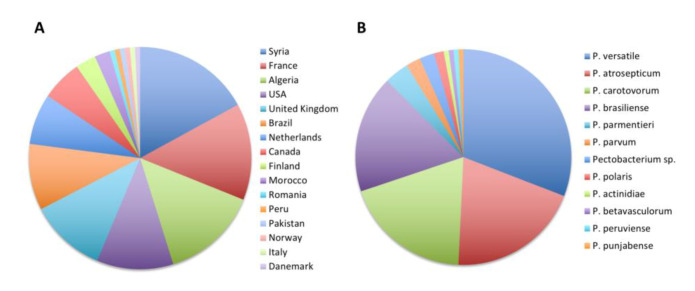
Updated taxonomy of the 136 strains isolated from potatoes. (**A**) Country of isolation and (**B**) proportion of strain isolated in each *Pectobacterium* species.

As already stated, a large majority of 136 strains were isolated from potato tubers or potato plants. These 136 strains were isolated in 16 countries covering four continents (Figure 5). We were therefore interested in understanding which species were isolated from this economically important crop. We found that strains isolated from potatoes belonged to 11 different already described *Pectobacterium* species (Figure 5). The most frequently deposited species was the recently described species *P. versatile* (42/136), followed by *P. atrosepticum* (27/136), *P. carotovorum* (26/136) and *P. brasiliense* (24/136). Less frequently, other recently described species known to infect potatoes, such as *P. parmentieri*, *P. polaris, P. parvum, P. peruviense* and *P. punjabense*, were also deposited to the collection (Figure 5 and Table 3). Surprisingly, our taxonomical update also identified one strain of *P. actinidiae* and one strain of *P. betavasculorum* that were isolated from potatoes in Syria in 2004 and Romania in 1992, respectively. Among the nonclassified strains, the three strains that grouped into a putative new species (CFBP 5380, CFBP 6067 and CFBP 6068) were isolated from potato plants and may represent a new pathogen species on this host plant. 

On potatoes, soft rot is the name usually used for tuber rotting, while the blackleg disease refers to the spread of the pathogen to the base of the potato stem, where it causes darkening and the decay of the aerial part [4]. Not all *Pectobacterium* spp. cause blackleg, but we could infer that a strain isolated from the stem or aerial part of the potato plant was isolated from blackleg disease symptoms. Out of the 136 strains isolated from potatoes, 31 were isolated from the stem or leaf and 33 from the tuber, and, unfortunately, it was not clearly documented for the remaining 71 strains (Table 3). The 31 strains isolated from the stem or leaf belonged to six species: five of which—*P. brasiliense, P. atrosepticum, P. parvum, P. parmentieri* and *P. punjabense*—are well-known species triggering blackleg disease [4,23,26]. Interestingly, the newly described *P. versatile* species was also isolated from potato stems or leaves in the UK, Morocco and France from 1972 to 2016. Whether *P. versatile* could be responsible for blackleg outbreak, or whether it is associated with blackleg symptoms as a secondary invader, remain to be determined. 

### 3.6. Analysis of Species Host Range and Geographic Distribution

The update of taxonomical assignation prompted us to check the host range and geographic distribution of species deposited at the CIRM-CFBP collection (Table 4). 

The *P. versatile* species is by far the most represented species of the CIRM-CFBP collection. The 72 *P. versatile* strains were isolated from 12 host plants representing nine botanical families and from water. *P. versatile* strains were isolated from 11 countries on three continents. This highlights the broad ecological and geographical distributions of this species. The most ancient *P. versatile* strain deposited in CIRM-CFBP was isolated in 1946. This contrasts with the recent description of this species in 2019. The reason why this species was so long-neglected probably comes from its close genomic proximity with *P. carotovorum* [15], and the taxonomical update performed here confirms that these two species were often mixed up by bacteriologists (Figure 4). *P. carotovorum* is the second-most represented species deposited in CIRM-CFBP, and, as *P. versatile*, it also has a broad ecological and geographical distribution (Table 4), also explaining why these two species were often mixed up. Another species with broad ecological and geographical distribution is *P. brasiliense* (Table 4). This is in contrast with *P. atrosepticum* strains, which were isolated from only one botanical family, *Solanaceae,* with 27 strains isolated from potatoes, 2 from tomatoes and 1 from the soil environment, certainly indicating a narrower ecological niche. The host range of *Pectobacterium* spp. was reviewed by Ma et al. in 2007 and updated by Charkowski in 2018 [3,4]. Our taxonomical update of the CIRM-CFBP extends the number of plant hosts from which *Pectobacterium* strains were isolated (Table 4, new plant hosts in bold). For example, we found that *P. brasiliense* and *P. versatile* could be isolated from *Chrysanthemum sp.,* an ornamental plant previously described as infected by *Dickeya* spp. and not by *Pectobacterium* spp. [3]. As well, *P. brasiliense* was isolated from *Musa* sp., a plant also previously reported to be infected only by *Dickeya* sp. [3]. 

The recently described “*P. zantedeschiae*” [27] and *P. parvum* species [23] had only a few representatives in CIRM-CFBP (Table 4). Nevertheless, some of the strains were isolated more than 50 years ago. Indeed, the first *P. parvum* strain was isolated in 1969, and the three “*P. zandeteschiae*” strains were respectively isolated in 1960, 1964 and 1966. This indicates that both species were already present on their respective host plants well before their description. Interestingly, although most of the *P. parvum* strains were isolated from potato plants, the *P. parvum* strain of 1969 was isolated from sunflowers enlarging the host range of this species. For “*P. zantedeschiae*”, the three strains hosted at CIRM-CFBP were all isolated from *Araceae* plants, as the other strains described for this species [27]. Concerning the recently described freshwater environmental species *P. aquaticum* [17], *P. fontis* [21] and *P. polonicum* [25] and the strain CFBP 8739, we were surprised that these species had either no representative in the CIRM-CFBP or only strains deposited following the recent description of the species. The fact that no strain representative of these species was isolated from crop plants and ornamentals along the 75-year period that covered our analysis strongly suggests that these species are not an important threat for crop plants and ornamentals. Whether these species evolved toward saprophytism and are no longer able to damage living plants or whether they infect living plants with no economic value remains to be determined. 

## 4. Conclusions

Bacterial culture collections hold resources that are diverse and can help scientists to better understand, in the case of pathogens, the history of the epidemics, the emergence of diseases and the host range of pathogenic bacteria. However, even if the situation improved a lot during the last decade, the quality of associated data to deposited resources is often scarce for old resources and can hamper the benefit that could be gained from bacterial collections.

By this publication, we wanted to demonstrate how efforts made by the collections to cure their resources are beneficial to the whole scientist community and bring a better understanding of the dynamic of the taxa considered. We strongly encourage culture collections to initiate or continue their efforts to update the identity of their resources.

We also encourage scientists to deposit their resources in public culture collections, where they will be made available long-term for the benefit of the whole scientific community. The quality of the resources being dependent on the quality of the associated data, the depositors are also strongly encouraged to transmit the most accurate and complete data, even if these data do not appear to be important at the time of deposit. Indeed, while the plant species or environment from which strains have been isolated is generally indicated, the type of symptoms is often missing, and for environmental strains isolated from water, the water temperature is often not indicated. As well, the year of isolation is important data that is sometimes neglected.

In the case of *Pectobacterium* spp., our work permitted a better overview of the extent of the diversity in the collection, uncovering potential new species and giving insights into the epidemiology and ecology of this genus.

## Figures and Tables

**Table 1:** General information for the 15 sequenced *Pectobacterium* genomes.

Strain	Accession	Size	Scaffolds	%GC	N50	Coverage	CDS	RNA
Psp CFBP8736	JACDSF000000000	4,665,864	169	51.2	107,870	118	4.322	52
Pb CFBP7357	JACDSB000000000	4,805,970	50	51.8	231,494	143	4.236	62
Pb CFBP3230	JACDSD000000000	4,850,518	50	52.1	394,958	179	4.243	62
Pb CFBP5381	JACDSC000000000	4,600,084	60	52.0	230,522	126	4.059	62
Pc CFBP6074	JACDRY000000000	4,691,819	92	52.0	207,976	156	4.171	62
Pc CFBP8734	JACDSA000000000	4,702,111	58	51.9	325,663	216	4.190	55
Pc CFBP1402	JACDRZ000000000	4,667,513	62	52.0	312,332	177	4.146	58
Pc CFBP7347	JACDRX000000000	4,694,461	42	52.0	317,373	189	4.148	63
Po CFBP8735	JACDRW000000000	4,974,102	129	51.4	131,982	203	4.466	71
Psp CFBP8739	JACDRR000000000	4,359,644	64	51.7	341,883	149	3.874	57
Pv CFBP1118	JACDRV000000000	4,968,197	210	51.9	51,811	180	4.419	41
Pv CFBP6698	JACDRS000000000	4,963,578	87	50.8	120,001	124	4.401	61
Pv CFBP5378	JACDRU000000000	4,942,943	57	51.8	277,607	154	4.338	61
Pv CFBP5669	JACDRT000000000	4,960,228	45	51.7	245,703	150	4.367	61
Pa CFBP8737	JACDSE000000000	4,898,716	50	51.8	326,273	178	4.357	60

Psp: P. unassigned species, Pb: P. brasiliense, Pc: P. carotovorum, Po: P. odoriferum, Pv: P. versatile and Pa: P. aroidearum.

**Table 2:** Pairwise average nucleotide identity (ANI) (below diagonal) and digital DNA-DNA hybridisation (dDDH) (above diagonals) for 6 of the analysed genomes.

		CFBP7357	CFBP5381	CFBP3230	PBR1692T	CFBP8736	PC1	CFBP8737	CFBP8739
Pb	CFBP7357	1.000	0.772	0.766	0.678	0.647	0.416	0.417	0.415
	CFBP5381	0.973	1.000	0.765	0.683	0.653	0.416	0.415	0.417
	CFBP3230	0.973	0.973	1.000	0.684	0.649	0.415	0.415	0.414
	PBR1692T	0.962	0.962	0.962	1.000	0.611	0.413	0.412	0.410
Psp	CFBP8736	0.955	0.957	0.956	0.947	1.000	0.412	0.409	0.407
Pa	PC1	0.905	0.906	0.905	0.904	0.903	1.000	0.832	0.432
	CFBP8737	0.906	0.906	0.905	0.904	0.904	0.980	1.000	0.429
Psp	CFBP8739	0.905	0.905	0.905	0.903	0.903	0.911	0.910	1.000

*Pb: P.brasiliense*. Pa: *P. aroidearum*. Psp: *Pectobacterium* unassigned species. The genomes analysed in this study are indicated in black. The reference genomes are indicated in red. ANI values and dDDH values above the threshold of 0.96 and 0.70 are indicated with a yellow background. Values inferiors to these thresholds are indicated with a blue background.

**Table 3:** Species isolated from potato plants and reported symptoms.

	nb of Strains Isolated on Potatoes/Species			
	Stems or Leaves	Tubers	Not Reported	Total
*P. versatile*	6	7	29	42
*P. atrosepticum*	6	11	10	27
*P. carotovorum*	0	4	22	26
*P. brasiliense*	14	7	3	24
*P. parmentieri*	2	3	0	5
*P. sp.*	0	1	2	3
*P. parvum*	2	0	1	3
*P. polaris*	0	0	2	2
*P. punjabense*	1	0	0	1
*P. peruviense*	0	0	1	1
*P. betavasculorum*	0	0	1	1
*P. actinidiae*	0	0	1	1
Total	31	33	72	136

**Table 4:** Host plant, environment and country of isolation for each of the *Pectobacterium* species deposited at the CIRM-CFBP.

Species	nb of Strains	Isolated from (nb of Strains)	Country of Isolation (nb of Strains)
P. versatile *	72	*Solanum tuberosum* (42) ***Cyclamen* sp. (4)** ***Chrysanthemum* sp. (3)** *Cichorium intybus* (3) ***Iris* (3)** ***Primula sp.* (3)** ***Allium porrum* (2)** *Brassica oleracea* (2) *Cynara scolymus L.* **(1)** ***Daucus carota* (1)** ***Hyacinthus orientalis* (1) ** Rhizosphere - ***Solanum dulcamara*** (1) freshwater (6)	Algeria (3) Canada (3) Finland (1) France (35) La Réunion ^#^ (1) Morocco (3) Netherland (8) Spain (1) Syria (6) UK (7) USA (4)
P. carotovorum *	38	***Aloe arborescens* (1)** ***Apium graveolens* (1)** *Brassica oleracea (*1) ***Capsicum annuum* (1)** ***Cyclamen* sp. (5)** *Solanum tuberosum* (26) freshwater (3)	Algeria (3) Canada (4) Denmark (1) France (5) Greece (2) Spain (2) Syria (11) USA (7) Yugoslavia (3)
*P. brasiliense*	34	***Carica papaya* (1)** ***Chrysanthemum* sp. (2)** *Cucurbita pepo* (1) ***Cyclamen* sp. (1)** ***Gossypium* sp. (1)** ***Musa* sp. (1)** *Solanum tuberosum* (24) Rhizosphere ***Solanum dulcamara*** (1) freshwater (2)	Algeria (7) Brazil (13) France (4) Italy (1) La Réunion ^#^ (1) Martinique ^#^ (1) Spain (2) Syria (3) USA (2)
*P. atrosepticum*	30	***Lycopersicon esculentum* (2)** *Solanum tuberosum* (27) soil (1)	Algeria (6) Canada (1) France (11) Italy (1) Syria (1) UK (7) USA (3)
*P. betavasculorum*	19	*Beta vulgaris* (15) ***Helianthus annuus* (2)** ***Opuntia phaeacantha* (1)** ***Solanum tuberosum* (1)**	France (9) Mexico (1) Romania (1) USA (7) Unknown (1)
*P. odoriferum*	19	***Allium porrum* (3)** *Apium graveolens* (1) ***Beta vulgaris* (1)** *Cichorium intybus* (11) ***Hyacinthus* sp. (2)** freshwater (1)	France (17) Unknown (2)
*P. aquaticum*	8	freshwater (8)	France (8)
*P. aroidearum*	7	***Lycopersicon esculentum* (1)** ***Nicotiana tabacum* (1) ** ***Philodendron floridi* (1) ** *Zantedeschiae* (3) freshwater (1)	France (4) Guadeloupe ^#^ (1) South Africa (1) USA (1)
*P. sp.*	7	*Solanum tuberosum* (3) *Musa* sp. (1) *Nicotinan tabacum (*1) freshwater (2)	USA (3) France (2) Martinique ^#^ (1) Netherland (1)
*P. cacticida*	6	***Acanthocereus pentagonus* (1)** *Carnegiea gigantea* (3) ***Lemaireocereus thurberi* (1)** ***Opuntia fulgida* (1)**	USA (6)
*P. parmentieri*	5	*Solanum tuberosum* (5)	UK (2) Netherlands (1) France (1) Finland (1)
*P. wasabiae*	5	*Eutrema wasabi* (syn *Eutrema japonicum*) (5)	Japan (5)
P. parvum **	4	*Solanum tuberosum* (3) *Helianthus annuus* (1)	Yugoslavia (1) Netherlands (1) Finland (2)
P. peruviense **	3	*Solanum tuberosum* (1) freshwater (2)	France (2) Peru (1)
P. zantedeschiae **	3	*Zantedeschia* sp. (2), *Arum* sp. (1)	France (3)
P. polaris **	2	*Solanum tuberosum* (2)	Syria (1) Norway (1)
*P. actinidiae*	1	***Solanum tuberosum*** **(1)**	Syria (1)
P. fontis **	1	freshwater (1)	Malaysia (1)
P. punjabense **	1	*Solanum tuberosum* (1)	Pakistan (1)

Plant indicated in bold correspond to plants not described as a host plant for the indicated species by Charkowski 2018. ^#^ French overseas territories. * As *P. carotovorum* and *P. versatile* were often mixed up, new host plants indicated in bold correspond to host plants not described as *P. carotovorum* host plants by Charkowski 2018. ** *Pectobacterium* spp. whose host range was not described by Charkowski 2018. The number of isolated strains on each host or country is indicated between parentheses.

## Supplementary Materials

The following are available online at https://www.mdpi.com/2076-2607/8/9/1441/s1: Two supplementary tables (Table S1 and Table S2) and one supplementary figure (Figure S1) are available with the online version of this article.
