# Supplementary material for: Updated Taxonomy of Pectobacterium Genus in the CIRM-CFBP Bacterial Collection: When Newly Described Species Reveal “Old” Endemic Population
Source: Microorganisms. 2020 Sep 20;8(9):1441. doi: 10.3390/microorganisms8091441 (PMC7565848; doi:10.3390/microorganisms8091441)
Supplement: Supplementary file 1 [file microorganisms-08-01441-s001.zip › supplementals/figS1.pdf]

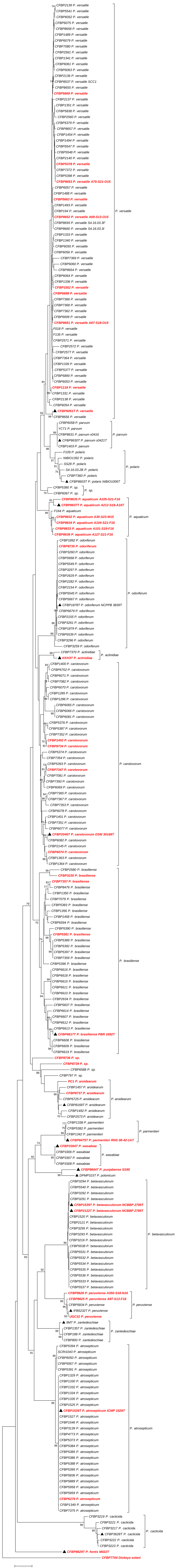
P. versatile
P. parvum
P. polaris
P. sp.
P. aquaticum
P. odoriferum
P. actinidiae
P. carotovorum
P. brasiliense
P. aroidearum
P. parmentieri
P. wasabiae
P. betavasculorum
P. peruviense
P. zantedeschiae
P. atrosepticum
P. cacticida

Figure S1: Extended view of the phylogenetic tree displayed Fig 1. Phylogenetic tree reconstructed from concatenated partial sequences from *dnaX*, *leuS* and *recA* housekeeping genes. The strains indicated in red correspond to strains whose genomes are deposited in NCBI database, the genomes references are provided Table S1. The phylogenetic tree was reconstructed with concatenated alignments of all genes with MEGA 7.0.26, using the neighbour-joining method with 1000 bootstrap replicates, and the evolutionary distances were computed by using the Kimura two-parameter method. Bootstrap values are shown when over 70%
