# Supplementary material for: Updated Taxonomy of Pectobacterium Genus in the CIRM-CFBP Bacterial Collection: When Newly Described Species Reveal “Old” Endemic Population
Source: Microorganisms. 2020 Sep 20;8(9):1441. doi: 10.3390/microorganisms8091441 (PMC7565848; doi:10.3390/microorganisms8091441)
Supplement: Supplementary file 1 [file microorganisms-08-01441-s001.zip › supplementals/Table S2 - 23 Juillet.docx]

|  |  | CFBP1402 | CFBP8734 | CFBP6074 | DSM30168 T | CFBP7347 | CFBP6051 T | CFBP5378 | CFBP1118 | CFBP5669 | CFBP6698 | NCPPB3839 T | CFBP8735 | CFBP7357 | CFBP5381 | CFBP3230 | PBR1692 T | CFBP8736 | PC1 | CFBP8737 | CFBP8739 |
| --- | --- | --- | --- | --- | --- | --- | --- | --- | --- | --- | --- | --- | --- | --- | --- | --- | --- | --- | --- | --- | --- |
| Pc | CFBP1402 | 1.000 | 0.993 | 0.853 | 0.773 | 0.770 | 0.641 | 0.640 | 0.634 | 0.635 | 0.635 | 0.615 | 0.610 | 0.518 | 0.518 | 0.514 | 0.509 | 0.503 | 0.402 | 0.399 | 0.401 |
|  | CFBP8734 | 0.999 | 1.000 | 0.846 | 0.768 | 0.764 | 0.638 | 0.636 | 0.632 | 0.631 | 0.632 | 0.610 | 0.607 | 0.521 | 0.521 | 0.517 | 0.512 | 0.505 | 0.403 | 0.399 | 0.401 |
|  | CFBP6074 | 0.983 | 0.982 | 1.000 | 0.775 | 0.769 | 0.644 | 0.639 | 0.635 | 0.635 | 0.637 | 0.612 | 0.610 | 0.519 | 0.517 | 0.515 | 0.509 | 0.504 | 0.402 | 0.400 | 0.400 |
|  | DSM30168^T^ | 0.974 | 0.974 | 0.974 | 1.000 | 0.835 | 0.641 | 0.639 | 0.637 | 0.635 | 0.632 | 0.615 | 0.611 | 0.521 | 0.519 | 0.518 | 0.510 | 0.501 | 0.403 | 0.401 | 0.398 |
|  | CFBP7347 | 0.974 | 0.973 | 0.973 | 0.981 | 1.000 | 0.641 | 0.638 | 0.636 | 0.634 | 0.633 | 0.618 | 0.615 | 0.518 | 0.519 | 0.517 | 0.508 | 0.501 | 0.403 | 0.400 | 0.399 |
| Pv | CFBP6051^T^ | 0.956 | 0.955 | 0.956 | 0.955 | 0.956 | 1.000 | 0.800 | 0.794 | 0.807 | 0.806 | 0.608 | 0.606 | 0.503 | 0.501 | 0.498 | 0.494 | 0.492 | 0.396 | 0.395 | 0.394 |
|  | CFBP5378 | 0.954 | 0.954 | 0.955 | 0.955 | 0.954 | 0.976 | 1.000 | 0.834 | 0.844 | 0.840 | 0.612 | 0.613 | 0.499 | 0.498 | 0.500 | 0.491 | 0.489 | 0.397 | 0.397 | 0.394 |
|  | CFBP1118 | 0.954 | 0.954 | 0.954 | 0.954 | 0.955 | 0.976 | 0.981 | 1.000 | 0.828 | 0.820 | 0.613 | 0.611 | 0.499 | 0.497 | 0.497 | 0.491 | 0.486 | 0.398 | 0.397 | 0.395 |
|  | CFBP5669 | 0.954 | 0.954 | 0.954 | 0.954 | 0.954 | 0.977 | 0.982 | 0.980 | 1.000 | 0.847 | 0.610 | 0.610 | 0.498 | 0.497 | 0.499 | 0.490 | 0.488 | 0.396 | 0.396 | 0.394 |
|  | CFBP6698 | 0.954 | 0.954 | 0.954 | 0.954 | 0.954 | 0.977 | 0.981 | 0.979 | 0.982 | 1.000 | 0.609 | 0.606 | 0.496 | 0.498 | 0.500 | 0.492 | 0.486 | 0.397 | 0.397 | 0.394 |
| Po | NCPPB3839^T^ | 0.951 | 0.950 | 0.950 | 0.951 | 0.952 | 0.950 | 0.950 | 0.950 | 0.950 | 0.949 | 1.000 | 0.930 | 0.480 | 0.481 | 0.481 | 0.474 | 0.469 | 0.395 | 0.394 | 0.391 |
|  | CFBP8735 | 0.950 | 0.950 | 0.950 | 0.951 | 0.951 | 0.949 | 0.950 | 0.949 | 0.950 | 0.949 | 0.991 | 1.000 | 0.479 | 0.479 | 0.481 | 0.475 | 0.465 | 0.394 | 0.393 | 0.389 |
| Pb | CFBP7357 | 0.933 | 0.934 | 0.933 | 0.933 | 0.933 | 0.929 | 0.929 | 0.928 | 0.928 | 0.927 | 0.923 | 0.923 | 1.000 | 0.772 | 0.766 | 0.678 | 0.647 | 0.416 | 0.417 | 0.415 |
|  | CFBP5381 | 0.932 | 0.933 | 0.933 | 0.933 | 0.933 | 0.929 | 0.928 | 0.927 | 0.928 | 0.928 | 0.923 | 0.923 | 0.973 | 1.000 | 0.765 | 0.683 | 0.653 | 0.416 | 0.415 | 0.417 |
|  | CFBP3230 | 0.932 | 0.933 | 0.932 | 0.933 | 0.932 | 0.928 | 0.929 | 0.928 | 0.928 | 0.928 | 0.923 | 0.923 | 0.973 | 0.973 | 1.000 | 0.684 | 0.649 | 0.415 | 0.415 | 0.414 |
|  | PBR1692^T^ | 0.930 | 0.932 | 0.930 | 0.931 | 0.931 | 0.927 | 0.927 | 0.926 | 0.927 | 0.926 | 0.921 | 0.921 | 0.962 | 0.962 | 0.962 | 1.000 | 0.611 | 0.413 | 0.412 | 0.410 |
| Psp | CFBP8736 | 0.929 | 0.929 | 0.929 | 0.928 | 0.928 | 0.926 | 0.925 | 0.924 | 0.924 | 0.924 | 0.919 | 0.919 | 0.955 | 0.957 | 0.956 | 0.947 | 1.000 | 0.412 | 0.409 | 0.407 |
| Pa | PC1 | 0.901 | 0.901 | 0.901 | 0.901 | 0.901 | 0.899 | 0.899 | 0.899 | 0.899 | 0.899 | 0.898 | 0.898 | 0.905 | 0.906 | 0.905 | 0.904 | 0.903 | 1.000 | 0.832 | 0.432 |
|  | CFBP8737 | 0.900 | 0.901 | 0.901 | 0.901 | 0.901 | 0.899 | 0.899 | 0.899 | 0.899 | 0.899 | 0.897 | 0.898 | 0.906 | 0.906 | 0.905 | 0.904 | 0.904 | 0.980 | 1.000 | 0.429 |
| Psp | CFBP8739 | 0.901 | 0.901 | 0.900 | 0.900 | 0.901 | 0.898 | 0.898 | 0.898 | 0.898 | 0.898 | 0.897 | 0.896 | 0.905 | 0.905 | 0.905 | 0.903 | 0.903 | 0.911 | 0.910 | 1.000 |

Table S2 : ANI (below diagonal) and dDDH (above diagonal) for the 15 analyzed genomes (indicated in black) and reference genomes (indicated in red).

Pc : *P. carotovorum*. Pv : *P. versatile*. Po : *P. odoriferum*. Pb : *P. brasiliense*. Pa : *P. aroidearum*. Psp : *Pectobaterium* unassigned species.
